# Cancer Stem-Like Cells in a Case of an Inflammatory Myofibroblastic Tumor of the Lung

**DOI:** 10.3389/fonc.2020.00673

**Published:** 2020-05-15

**Authors:** Valentina Masciale, Giulia Grisendi, Federico Banchelli, Roberto D'Amico, Antonino Maiorana, Pamela Sighinolfi, Lucio Brugioni, Alessandro Stefani, Uliano Morandi, Massimo Dominici, Beatrice Aramini

**Affiliations:** ^1^Division of Thoracic Surgery, Department of Medical and Surgical Sciences, University of Modena and Reggio Emilia, Modena, Italy; ^2^Division of Oncology, Department of Medical and Surgical Sciences, University of Modena and Reggio Emilia, Modena, Italy; ^3^Center of Statistic, Department of Medical and Surgical Sciences, University of Modena and Reggio Emilia, Modena, Italy; ^4^Department of Medical and Surgical Sciences, Institute of Pathology, University of Modena and Reggio Emilia, Modena, Italy; ^5^Internal Medicine and Critical Care Unit, Department of Integrated Medicine, Emergency Medicine and Medical Specialties, University of Modena and Reggio Emilia, Modena, Italy

**Keywords:** inflammatory myofibroblastic tumor of the lung, cancer stem cells, cancer stem-like cells, mitosis, target therapy

## Abstract

**Background:** Inflammatory myofibroblast tumor (IMT) is a rare tumor with obscure etiopathogenesis in which different inflammatory cells and myofibroblastic spindle cells are seen histologically. Although the majority of these neoplasms have a benign clinical course, the malignant form has also been reported. The gold standard is surgical treatment for complete removal. Our report describes a 50-year-old woman who underwent surgery for IMT of the lung. The aim is to determine whether cancer stem cells may be present in IMT of the lung.

**Methods:** In April 2018, the patient underwent surgery for tumor mass asportation through lateral thoracotomy. The histology of the tumor was consistent with IMT of the lung. The ALDEFLUOR assay, after tissue digestion, was used to identify and sort human lung cancer cells expressing high and low aldehyde dehydrogenase (ALDH) activity. SOX2, NANOG, OCT-4, and c-MYC positivity were additionally determined by immunohistochemistry.

**Results:** The specimen contained 1.10% ALDH^high^ cells among all viable lung cancer cells, which indicates the population of cancer stem cells is not negligible. Immunohistochemically assessed cell positivity for ALDH1A1, SOX2, NANOG, OCT-4, and c-MYC, which are considered as lung cancer stem-like cells markers.

**Conclusion:** For the first time, we demonstrated the presence of cancer stem cells in a case of IMT of the lung. This finding may provide a base for considering new pathological and molecular aspects of this tumor. This perspective suggests further studies to understand the possibility of developing recurrence depending on the presence of cancer stem cells.

## Introduction

The inflammatory myofibroblastic tumor (IMT), first described by Brunn in 1937, is an extremely rare type of inflammatory pseudo-tumor. The prevalence is between 0.04 and 0.7%, independent of gender and race (1–3). It is debated whether an IMT is a benign or malignant lesion and this is often challenging for further clinical decisions. However, the lungs are considered the most common site for the presentation of this tumor (1).

There are cases of recurrence described in the literature, not only in the lungs but also in other organs, however the recurrence rate at 10 year survival is lower than 90% (4, 5). Beside this aspect, the pathological nature of this tumor is still debated.

In 2002 the World Health Organization classified IMT as an intermediate grade malignancy (6). One of the most recent discoveries is related to chromosomal translocation involving the ALK gene, which seems to be present in 50% of cases with malignant characteristics (7). The treatment of choice for IMTs is surgical resection in order to guarantee a favorable prognosis (4).

Our work aims to detect the presence of cancer stem-like cells (CSCs) in a case of IMT of the lung by immunohistochemical testing for the most common CSCs markers, with aldehyde dehydrogenase (ALDH), as well as for the presence of pluripotent transcription factors such as OCT4, SOX-2, NANOG, and C-MYC, which modulate biological CSC activities (8–10). This will provide a base for further studies with a larger cohort of patients considering the presence of CSCs as a new pathological marker and a possible predictive factor of aggressiveness in this type of tumor.

## Methods

### Case Presentation

**Figure 1:**
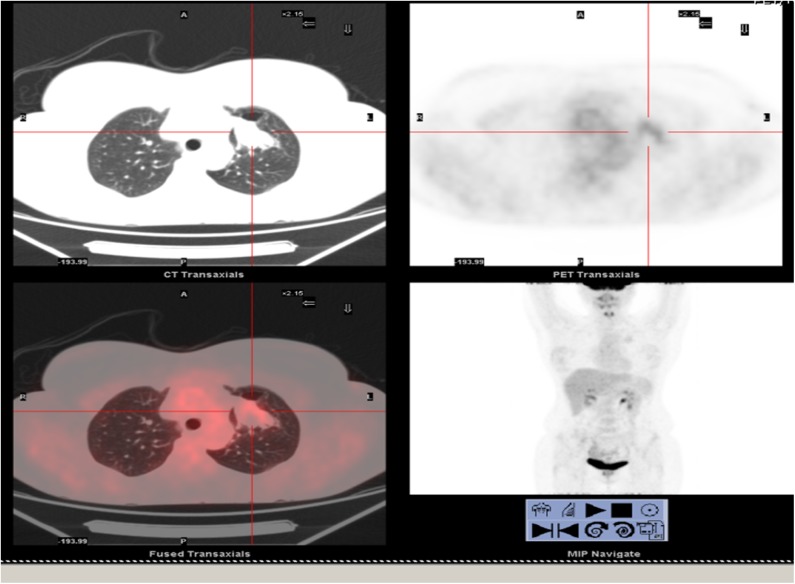
Total body PET/CT scan. Lung nodule at the upper left lobe showed a very low uptake of the lesion.

In April 2018, a 47-year-old woman came to our attention for dyspnea and tachycardia under exertion. An x-ray of the chest showed a large mass in the left hemithorax. A CT scan with enhancement presented a 40 ×30 cm mass involving the left upper lobe of the lung. A CT guided biopsy gave a diagnosis of benign lung tumor. An Emission Tomography – Computed Tomography (PET/CT) scan showed a very mild uptake at the level of the nodule (SUV max = 2.3) with no other signs of uptake in other parts of the body (Figure 1).

For the symptomatology and the dimension of the mass, the patient underwent a left upper lobectomy through lateral thoracotomy. The final histology showed an IMT of the lung. The cells were positive for actin in smooth muscle, although negative for ALK, MNF116, and estrogenic receptors, as well as for tuberculosis.

Patient underwent a clinical check ten days after surgery by the oncologist who suggested a period of follow up every 6 months for the first and second year from surgery, and every year after the second year, for a total of 5 years of radiological and clinical monitoring.

### Cells Extraction and FACS Analysis

Sterile Dulbecco's PBS (L1825-BC—Merck Millipore) was used to wash the IMT tissue, then minced mechanically into millimetric pieces, and further digested using MACS™ C-Tube (Miltenyi) tumor dissociation kit, according to the manufacturer's instructions. The tissue was digested for 60 min at 37°C, filtered through a sterile cell strainer and centrifuged at 300 × g for 5 min, then resuspended in a DMEM and HAM'S F12 media mixture (2:1) (Gibco) containing penicillin-streptomycin and glutamine. The primary single-cell suspension was diluted in an ALDEFLUOR buffer containing BODIPY-aminoacetaldehyde (STEMCELL Technologies, Vancouver, BC). The morphology of the cell population was studied using side scatter (SSC) and forward scatter (FSC). Dead cells were identified and eliminated using 7-AAD (7-amino-actinomycin D) staining. Cell sorting and ALDH analysis were performed using a FACS-ARIA III (Becton Dickinson, Franklin Lakes, NJ). Results were analyzed by fluorescence-activated cell sorting (FACS) Diva software (Becton Dickinson). ALDH^high^ gate was included as gating strategy (11–13).

### Immunohistochemistry

The patient's slides were deparaffinized, rehydrated and then washed in PBS. Sodium citrate buffer was used for antigen retrieval. Samples were incubated with anti- ALDH1A1 (1:100) (Abcam, Cambridge, UK), anti-SOX2 (1:200) (MA1-014 Thermo Fisher Scientific, Meridian Road Rockford, IL, USA), anti-NANOG (1:200) (Thermo Fisher Scientific, Meridian Road Rockford, IL, USA), anti-OCT-4 (Cell Marque, Sierra College Blvd. Rocklin, California United States and anti-c-MYC (Ventana Medical Systems, Tucson, Arizona, USA) overnight at 4°C. Images were collected and the positivity was evaluated with Zeiss AxioCam ICc 3 High-Resolution through an Axioskop microscope camera. Section samples were investigated to evaluate the immunoreactivity to the markers used. A semi-quantitative method based on the evaluation of the positivity of the tumor cells was used. Here are shown the score classes: 0 (<5% positive), 1 (5 to 25% positive), 2 (>25 to 50% positive), 3 (>50 to 75% positive), and 4 (>75% positive) (14). Sections were scored by two trained investigators, blinded to patient's outcome and other clinical findings.

## Results

### Clinical Setting

The patient was discharged after six days from surgery with no complications. A chest x-ray after one week was normal following the operation. The patient underwent follow up and no recurrence was observed within a year after surgery.

### ALDH^high^ Stem Cells Were Identified in Primary Cells of an IMT of the Lung

**Figure 2:**
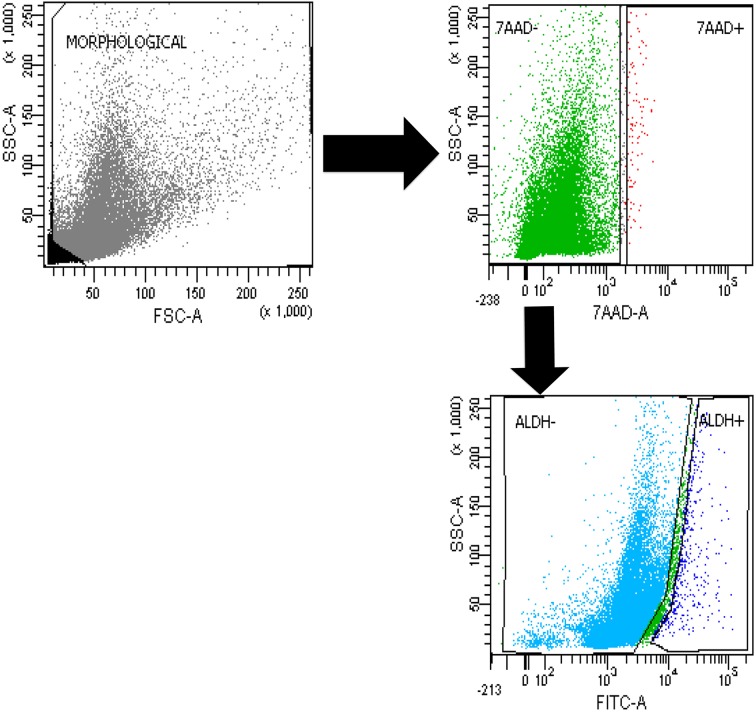
FACS analysis. Cytofluorimetric analysis of ALDH^high^ cells in a case of lung myofibroblastic tumor.

Tumor tissue dissociation efficiently released cancer cells characterized by a heterogeneous morphology, as illustrated in the widespread FSC and SSC values (Figure 2) (11–13). The mean viability of the samples was 99.7% based on 7-AAD staining. These data further confirmed that the developed dissociation procedure was a non-toxic approach to isolating cells from tumor tissues. The CSCs were physically separated from the bulk parental tumor cells and recovered by FACS according to the following gating strategy. Tumor cells were first identified based on their morphological parameters (FSC/SSC), and the ALDH activity was measured in the 7-AAD negative cell population only (Figure 2). ALDH^low^ and ALDH^high^ cells were both selected and sorted (Figure 2). An ALDH^high^ subpopulation accounted for 1.10% of all viable lung cancer cells, which indicates that a non-negligible population of tumor cells had characteristics of CSCs.

### Immunohistochemical Evaluation of the ALDH1A1, SOX2, NANOG, OCT-4, and c-MYC Stem Cell Markers in an IMT of the Lung

**Figure 3:**
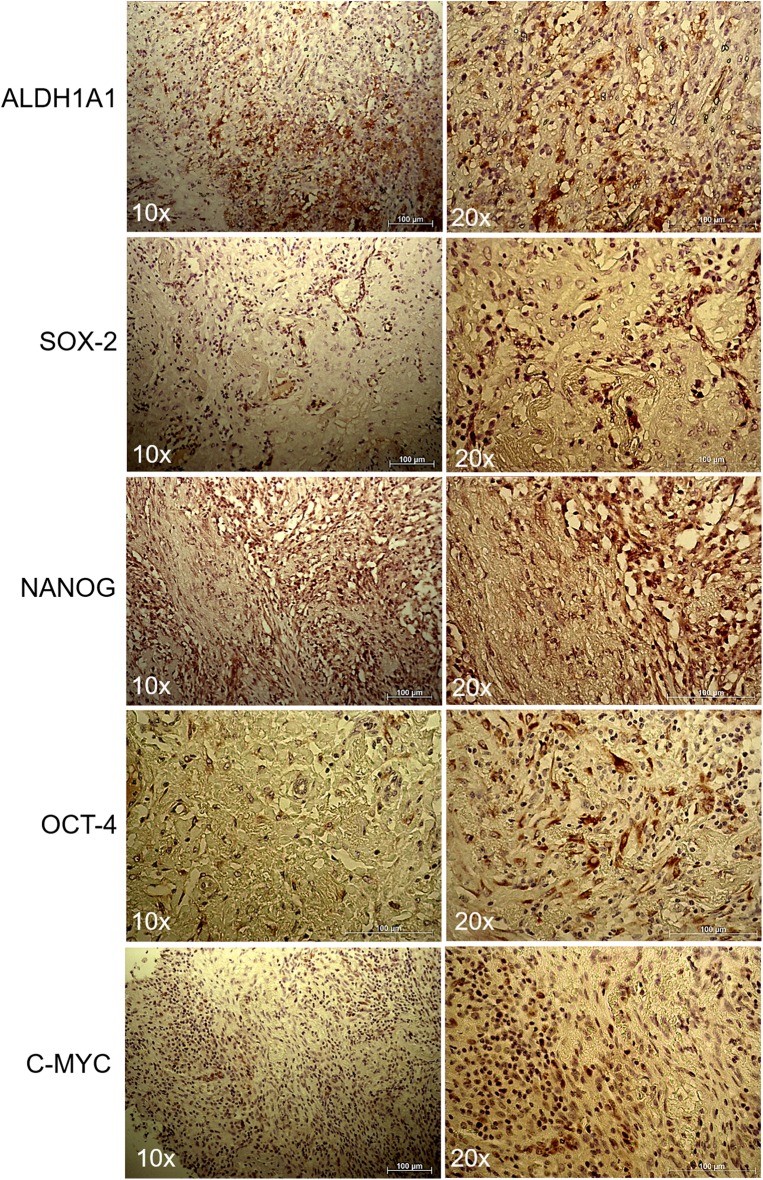
Immunohistochemistry of lung myofibroblastic tumor. Representative immunohistochemical staining of ALDH1A1, SOX2, NANOG, OCT-4, and c-MYC stem cell markers on a myofibroblastic tumor. Images were shown at 10x and 20x magnification.

To further evaluate the stemness of the cells extracted from the tumor biopsy, SOX2 immunohistochemistry was performed. Positive cells from tissue slides, represented by brown nuclei, were analyzed at 10x and 20x magnification. As expected from the FACS analysis, a non-negligible percentage of the cells were positive for ALDH1A1, SOX2, NANOG, OCT-4, and c-MYC (Figure 3).

## Discussion

In our report we first described the presence of cancer stem-like cells in a case of IMT of the lung. The connection between benign and malignant lesions has been investigated for a long time (15–17). Besides the different causes of malignancy, infectious disease may be one of the main causes of induced tumorigenesis (18). In fact the inflammatory microenvironment seems to be the key for the development and maintenance of the CSCs' niche (19). These mechanisms can be justified through the enhancement of proliferation, induction and metastatic signaling (15). In this report, our aim is to highlight the presence of cancer stem-like cells in combination with IMT inflammatory components. In particular, the possibility of emerging inflammatory mechanisms inducing stemness of CSCs has been previously described (19). In breast cancer, for example, the inflammatory factors are considered as one of the main causes of tumorigenesis (20, 21). However, the histogenesis of IMTs is unclear at the moment; some researchers suggest that IMTs are benign lesions with impacts for surgery, trauma, radiotherapy, steroids, and infectious agents, without convincing explanations for the entire scientific community (22–24). There are other hypothesis suggesting that IMTs are tumors related to genetic modifications, such as the recurrent involvement of chromosomal region 2p23, which seems to induce the aggressive local behavior and metastasis of this tumor (25). In particular, Coffin et al. demonstrated that some IMTs are neoplastic lesions with clonal aberrations (25). Additionally, it is very difficult to define histologically specific characteristics for this disease which can be used to define possible markers, and this is probably due to the impossibility of distinguishing heterogeneity in clinical aspects (26).

As a result, several teams tried to define lesions as falling under the category of IMT.

The World Health Organization has classified IMTs as a distinct entity, being tumors of intermediate biological potential for their capacity to generate local recurrence and, in some cases, distant metastasis (27).

Histologically IMTs are described as cellular or fascicular fibroblastic proliferations associated with chronic inflammatory infiltrate (27). It has been suggested that there are histologic criteria to define the malignant transformation as, for example, round cells associated with necrosis, large nucleoli, several mitoses, etc. (27). However, the main characteristic of these tumors is the *myofibroblast*, which justified the term of “inflammatory myofibroblastic tumors” (26). However, no attention until now has been given to the possible presence of cancer stem-cells in this tumor (27–30). Our study represents the first attempt at highlighting new cell populations that may be the key to better characterizing this type of tumor. In conclusion, we aim to determine new pathological markers as well as to aid the development of targeted therapies.

## Data Availability Statement

The datasets generated for this study are available on request to the corresponding author.

## Ethics Statement

The studies involving human participants were reviewed and approved by the Ethics Committee at University Hospital of Modena, MODENA, Italy, on 17 March 2017, Prot. N. 914/C.E and has been performed in accordance with the Declaration of Helsinki. The patients/participants provided their written informed consent to participate in this study.

## Author Contributions

The idea for the manuscript was conceived in September 2016 by BA and MD and was further developed by VM, GG, FB, RD'A, AM, LB, AS. AM and PS were involved in histopathological diagnosis. BA, VM, and FB wrote the first draft of the manuscript. BA and UM have been involved in surgery and tissue collection. VM and GG performed laboratory experiments, whereas FB and RD'A performed the statistical analysis. BA, VM, FB, MD, RD'A, AM, and UM reviewed and edited the manuscript before submission. All authors approved the final manuscript before the submission.

## Conflict of Interest

The authors declare that the research was conducted in the absence of any commercial or financial relationships that could be construed as a potential conflict of interest.

## Abbreviations

ALDH: aldehyde dehydrogenase
FACS: fluorescence-activated cell sorting
CSCs: cancer stem cells
SSC: side scatter
FSC: forward scattered
IMT: inflammatory myofibroblastic tumor.
